# Density, Destinations or Both? A Comparison of Measures of Walkability in Relation to Transportation Behaviors, Obesity and Diabetes in Toronto, Canada

**DOI:** 10.1371/journal.pone.0085295

**Published:** 2014-01-14

**Authors:** Richard H. Glazier, Maria I. Creatore, Jonathan T. Weyman, Ghazal Fazli, Flora I. Matheson, Peter Gozdyra, Rahim Moineddin, Vered Kaufman Shriqui, Gillian L. Booth

**Affiliations:** 1 Li Ka Shing Knowledge Institute, St. Michael's Hospital, Toronto, Ontario, Canada; 2 Institute for Clinical Evaluative Sciences, Toronto, Ontario, Canada; 3 Department of Family and Community Medicine, St. Michael's Hospital, Toronto, Ontario, Canada; 4 Dalla Lana School of Public Health, University of Toronto, Toronto, Ontario, Canada; 5 Department of Family and Community Medicine, University of Toronto, Toronto, Ontario, Canada; 6 Department of Medicine, University of Toronto, Toronto, Ontario, Canada; University of Sao Paulo, Brazil

## Abstract

The design of suburban communities encourages car dependency and discourages walking, characteristics that have been implicated in the rise of obesity. Walkability measures have been developed to capture these features of urban built environments. Our objective was to examine the individual and combined associations of residential density and the presence of walkable destinations, two of the most commonly used and potentially modifiable components of walkability measures, with transportation, overweight, obesity, and diabetes. We examined associations between a previously published walkability measure and transportation behaviors and health outcomes in Toronto, Canada, a city of 2.6 million people in 2011. Data sources included the Canada census, a transportation survey, a national health survey and a validated administrative diabetes database. We depicted interactions between residential density and the availability of walkable destinations graphically and examined them statistically using general linear modeling. Individuals living in more walkable areas were more than twice as likely to walk, bicycle or use public transit and were significantly less likely to drive or own a vehicle compared with those living in less walkable areas. Individuals in less walkable areas were up to one-third more likely to be obese or to have diabetes. Residential density and the availability of walkable destinations were each significantly associated with transportation and health outcomes. The combination of high levels of both measures was associated with the highest levels of walking or bicycling (p<0.0001) and public transit use (p<0.0026) and the lowest levels of automobile trips (p<0.0001), and diabetes prevalence (p<0.0001). We conclude that both residential density and the availability of walkable destinations are good measures of urban walkability and can be recommended for use by policy-makers, planners and public health officials. In our setting, the combination of both factors provided additional explanatory power.

## Introduction

Over the past two decades there has been a large increase in the prevalence of metabolic diseases such as obesity and diabetes in both developed and developing countries [1]–[4]. The etiology of obesity is multidimensional and includes environmental, genetic and behavioral factors which ultimately lead to an energy imbalance reflecting both increased caloric intake and decreased energy expenditure due to inadequate physical activity [1], [3], [5], [6]. While interventions to prevent obesity and promote healthy body weight are most often aimed at individuals, there is a growing recognition of upstream environmental factors, including the urban built environment, as potential targets for intervention [1], [7], [8].

More than half of the world's population now lives in cities and this trend is rapidly accelerating, underscoring the need to understand the health impacts of urban living [9]. A number of measures have shown associations between the built environment and transportation behaviours, physical activity, body mass index (BMI) or obesity [10]–[17]. Some studies have focused on individual built environment characteristics [17]–[22] while in others combinations of characteristics (e.g. “walkability” indices) have been developed and examined [23]–[28]. Indices that combine multiple aspects of the built environment often differ in composition and data sources, but most encompass some combination of: population or residential density; proximity of retail and service destinations; land use mix; and street connectivity. Residents of urban areas with combinations of high population density, many destinations, connected streets with short blocks, and a high land use mix tend to have higher rates of utilitarian or transport-related walking [16], [17], [25], [27], [28] and bicycling [29] and lower car use [20], [25], while individuals living in areas that lack those features are generally more car-dependent and have lower rates of active transportation.

Despite the rapid growth of built environment and health research over recent years, relatively few studies have considered the potential synergistic effects of density and destinations on walking behaviors and related health outcomes [30], [31]. From a planning and policy perspective it remains pertinent to consider which aspects of the built environment, both individually and in combination, enhance or impede utilitarian walking and which are most closely associated with health outcomes of relevance. The objective of this study was to examine which built environment characteristics were most strongly associated with active transportation behaviors, overweight, obesity and diabetes. Additionally, we sought to examine whether the additive effect of population or residential density and walkable destinations was more strongly associated with these outcomes than either measure on its own. Few studies have examined this type of interaction, yet built environment characteristics rarely exist in isolation on the ground.

We made use of a validated walkability index [32] that includes the components of density, destinations and street connectivity. This index has shown important associations with diabetes [33] but its components have not been examined separately. Although each of the components is known to capture important aspects of walkability [34], the focus of this work is on the interaction between density and destinations. Although an important feature of walkable environments, street connectivity is hard to modify in developed areas, while density and destinations are modifiable even in established communities through changes in zoning, urban planning and design.

## Methods

### Setting and Data Sources

This study combined multiple data sources in order to examine the association between the built environment, transportation behavior, and health outcomes between 2003 and 2009 among residents in the city of Toronto, Canada (2011 Canada census population of 2,615,060). We utilized built environment data from Statistics Canada (2006), DMTI Spatial Inc. Enhanced Points of Interest (2009), the City of Toronto (2009), and the Ministry of Education (2009) to create the walkability index components [32]. Self-reported transportation behaviors were derived from the 2006 Transportation Tomorrow Survey (TTS), available from the Data Management Group at the University of Toronto [35]. Self-reported weight and height were derived from the national Canadian Community Health Survey (CCHS), available from Statistics Canada [36]. Diabetes prevalence was derived from the Ontario Diabetes Database (ODD, 2009) using provincial administrative health claims data housed at the Institute for Clinical Evaluative Sciences [37]. The ODD is a validated population-based electronic database that identifies persons with diagnosed diabetes based on hospitalization and physician claims data [38]. The level of analysis was the dissemination block (DB) (N = 10,180), which is the smallest geographic unit for which census population and dwelling data are available. All analyses were conducted using SAS, version 9.3 and ArcGIS 10. Approval was granted by the Research Ethics Board of the Sunnybrook Health Sciences Centre.

### Walkability

The walkability measure used in this paper is an updated version of a composite walkability index that was developed and validated for Toronto and that has previously been used to examine relationships between walkability, immigration and diabetes incidence [33]. The method used for its development has been described in detail elsewhere [32]. The original version of the index used factor scores for weighting, whereas for simplicity in this study the components of the index were weighted equally. The current and previous version of the index are highly correlated (R = 0.997, p<0.0001) and have virtually identical geographic distributions in our setting.

The walkability index was comprised of four components, each calculated using 800m geographic buffers around a given DB's residentially-weighted centroid (geometric center point): (1) population density – calculated as the total number of people per square kilometer (from the 2006 Canada Census); (2) residential density – calculated as the total number of occupied residential dwellings per square kilometer (from the 2006 Canada Census); (3) availability of walkable destinations – calculated as the sum of all “retail and service” destinations including public recreation centers and schools (from DMTI Spatial Inc., 2009; City of Toronto, 2009; and Ministry of Education, 2009; see Text S1 for definitions); (4) street connectivity – calculated as the count of all intersections (from DMTI Spatial Inc., 2009) with at least 3 converging roads or pathways divided by the area of the buffer. The components were then equally weighted to create the final index measure. Quintiles of the walkability index and each of its components were generated by ordering DBs according to increasing walkability and allocating an equal number to each quintile.

### Active Transportation and Health Outcomes

To investigate whether the walkability index and its components were associated with levels of active transportation and ultimately with body weight and risk of diabetes, we compared the measures with self-reported survey data from the TTS and CCHS, and provincial administrative health claims from the ODD.

Active transportation-related outcomes extracted from the 2006 TTS (N = 51,612) included: average number of vehicles per household and average daily number of trips per person by walking or bicycling, public transit, and automobile. The TTS survey design and sampling frame are described elsewhere (Transportation Tomorrow Survey, 2008). Using CCHS data we calculated body mass index (BMI) as weight in kilograms divided by height in meters squared (weight [kg]/height [m^2^]) and determined the proportion of the population aged 30 to 64 who were ‘overweight or obese’ (BMI ≥25) or ‘obese’ (BMI ≥30) [39]. In order to increase the sample size for the CCHS data, we combined three CCHS cycles from 2003 to 2008 for a total of 9,757 respondents. Adjusted sampling weights for the combined sample were statistically computed and used for descriptive analyses. The ODD was used to determine the age-sex standardized prevalence of diabetes in 2009 of individuals aged 30 to 64 living in Toronto (N = 1,311,485).

In order to determine the level of walkability that individuals were exposed to in their residential neighborhoods, individuals from each of the above data sources were linked to their DB of residence using a Postal Code Conversion File [40]. Based on their DB of residence individuals were assigned a value, and a corresponding quintile, for the walkability index and each of its four individual components.

### Analysis

#### Agreement between walkability index, density and destinations

**Figure 1:**
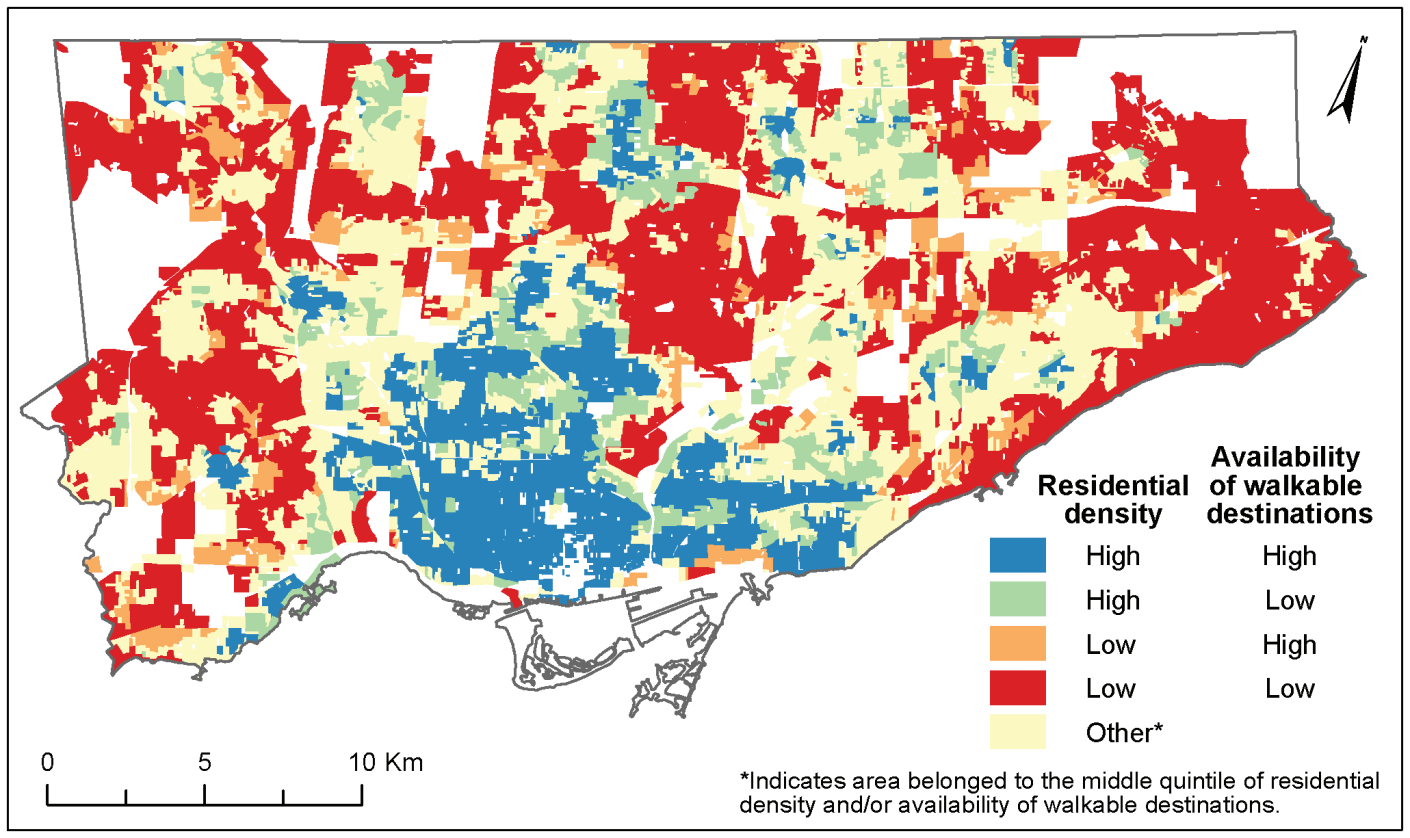
Spatial Concordance Between Residential Density and Availability of Walkable Destinations. Data from: the 2006 Canada Census; DMTI Spatial Inc., 2009; the City of Toronto, 2009; and the Ministry of Education, 2009. Residential density was calculated as the number of residential dwellings per square kilometer in all census disseminations blocks (DB) intersecting an 800 m buffer of a given DB's residentially-weighted centroid. Availability of walkable destinations was calculated as the number of retail and service destinations, including public recreation centers and schools, within an 800 m buffer of a given DB's residentially-weighted centroid. A DB was labeled ‘high’ for residential density or availability of walkable destinations if its value for that attribute placed it within the highest 2 quintiles for that attribute; similarly a DB was labeled as ‘low’ if its value placed it within the lowest 2 quintiles for that attribute.

Spearman rank correlation coefficients were used to measure agreement between the walkability index and each of its components at the DB level. To further explore the spatial correspondence between levels of density and availability of walkable destinations we created a 4-level variable representing combinations of high or low values for each attribute at the DB level of geography. A DB was labeled ‘high’ for residential density or availability of destinations if its value for that attribute placed it within the highest 2 quintiles for that attribute; similarly a DB was labeled as ‘low’ if its value placed it within the lowest 2 quintiles for that attribute. DBs whose value fell within the middle quintile were not included in this analysis. Initial analyses were performed using population density and residential density, however, we found that the measures were highly and significantly correlated (r = 0.96, p<0.0001) so we decided to use only one measure of density in this analysis. Residential density was selected as it is used more often in the walkability literature [23], [41]–[43]. Patterns of concordance between density and destinations were then depicted spatially on a map (Figure 1).

#### Association of walkability index and components with health data

Means and/or proportions were calculated for each outcome within each walkability index quintile. Rate ratios of the lowest (least walkable) to highest (most walkable) quintile along with 95% confidence intervals were also generated for each outcome. The same analyses were performed for each individual component of the walkability index. For the CCHS survey data, bootstrapping methods were used to estimate the standard error in order to calculate confidence intervals.

In order to better understand the potential synergistic effect of residential density and walkable destinations in relation to our transportation and health outcomes, interactions between those components and outcomes were further explored descriptively. Estimates for the individual outcomes were generated for each combination of residential density and walkable destinations quintiles and then plotted on a graph. Due to the relatively small number of obese individuals in our survey sample, further subdividing the sample for this analysis resulted in unstable estimates so we did not test for interactions with obesity. Statistical significance of the interaction terms was evaluated using general linear modeling.

## Results

The overall measure of walkability had moderately strong to strong correlations with each of its components: 0.72 with destinations, 0.88 with population density, 0.90 with residential density, and 0.78 with street connectivity. Destinations had only modest correlations with density measures (0.52 for population density and 0.56 for residential density) and with street connectivity (0.58), while population density and residential density were highly correlated (0.98). All correlations were statistically significant (p<0.0001).

The patterns seen in Figure 1 indicate a high degree of spatial concordance between residential density and the availability of destinations. Although some areas had high density and few destinations or vice versa, they were much less common than areas where there was concordance.

Characteristics of the study area by walkability quintiles are shown in Table 1. There were few clear socio-demographic differences across quintiles apart from a higher proportion of children and seniors living in the least walkable areas, and a higher average household income, yet lower education, in the least walkable areas. The variation in walkability components between the highest and lowest walkability areas was approximately four-fold for population density (ratio of Q1:Q5  = 0.24), five-fold for residential density (Q1:Q5  = 0.18), three-fold for street connectivity (Q1:Q5 ratio 0.35) and twenty five-fold for destinations (Q1:Q5  = 0.04).

**Table 1:** Sociodemographic* and Built Environment Characteristics by Walkability Index Quintile**.

	City of Toronto	Q1 (Lowest) Walkability)	Q2	Q3	Q4	Q5 (Highest) Walkability)	Q1:Q5 Ratio
**Sociodemographic Characteristics**							
**Total Population**	2,446,029	567,656	481,452	456,028	426,096	514,797	1.10
**Age (% of total)**							
**0**–**19**	21.7	23.0	22.7	22.9	22.5	17.2	1.33
**20**–**44**	36.3	32.3	33.1	34.1	36.6	45.7	0.71
**45**–**64**	26.6	27.7	26.9	26.7	27.0	24.9	1.11
**65+**	15.4	16.6	17.4	16.5	13.9	12.1	1.37
**Male (%)**	48.5	48.6	48.4	48.3	47.9	49.4	0.99
**Socioeconomic Status**							
**Average household income ($)**	70,133	76,258	73,457	69,002	72,469	59,232	1.29
**% population living below the low-income cut-off** ***	17.3	14.8	15.4	17.2	16.9	22.5	0.66
**Education**							
**University degree or higher (%)**	45.2	43.0	42.2	42.7	49.1	49.2	0.87
**Immigration**							
**% Immigrants**	43.0	47.2	46.2	45.0	37.1	39.3	1.20
**Built Environment Characteristics**							
**Population density (population/km^2^)**	4,828.0	2,198.7	3,186.3	4,146.3	5,665.5	8,982.8	0.24
**Dwelling density (dwellings/km^2^)**	2,118.8	805.3	1,217.6	1,645.6	2,495.6	4,450.6	0.18
**Street Connectivity (intersections/km^2^)**	27.6	14.7	21.8	26.8	33.0	41.8	0.35
**Walkable Destinations (within 800 m)**	35	4.9	9.1	15.7	35.1	109.2	0.04

*Sociodemographic data was derived from 2006 Canada Census dissemination area data for the City of Toronto overall.

**Built environment characteristics and the walkability index were calculated using data from: the 2006 Canada Census; DMTI Spatial Inc., 2009; City of Toronto, 2009; and the Ministry of Education, 2009.

***Before tax income was used for this measure.

As compared with individuals in the most walkable areas, those living in areas with the lowest walkability owned almost twice as many vehicles (Q1:Q5 ratio  = 1.80, 95% CI: 1.25–2.34), were almost twice as likely to travel by automobile (Q1:Q5 ratio  = 1.75, 95% CI: 1.20–2.30), were almost half as likely to use public transportation (Q1:Q5 ratio 0.58, 95% CI: 0.30–0.87) and roughly one-third as likely to walk or bicycle (Q1:Q5 ratio  = 0.32, 95% CI: 0.0–0.71) (Table 2). These results were consistent and significant for population density, residential density and availability of destinations, and were in the same direction but less strong for street connectivity.

**Table 2:** Transportation Behaviors* by Quintiles of Walkability and its Components**.

Quintile (1 = lowest; 5 = highest)	Average Daily Number of Trips per Person			Vehicles per Household
	Walk or Bicycle	Public Transit	Automobile	
**Walkability Index**				
**1**	0.10	0.36	1.22	1.33
**2**	0.11	0.37	1.14	1.24
**3**	0.12	0.41	1.08	1.14
**4**	0.16	0.50	1.02	1.02
**5**	0.30	0.62	0.70	0.74
**Q1:Q5 ratio (95% CI)**	0.32 (0.0–0.71)	0.58 (0.30–0.87)	1.75 (1.20–2.30)	1.80 (1.25–2.34)
**Walkability Index Components**				
**Population Density**				
**1**	0.10	0.35	1.31	1.37
**2**	0.11	0.37	1.20	1.28
**3**	0.12	0.40	1.10	1.18
**4**	0.15	0.47	1.00	1.04
**5**	0.27	0.61	0.71	0.75
**Q1:Q5 Ratio (95% CI)**	0.36 (0.0–0.83)	0.57 (0.27–0.88)	1.85 (1.27–2.42)	1.82 (1.28–2.36)
**Residential Density**				
**1**	0.10	0.34	1.28	1.43
**2**	0.11	0.36	1.20	1.28
**3**	0.12	0.40	1.08	1.15
**4**	0.15	0.48	1.01	1.05
**5**	0.29	0.62	0.72	0.75
**Q1:Q5 Ratio (95% CI)**	0.35 (0.0–0.78)	0.55 (0.26–0.84)	1.76 (1.22–2.31)	1.90 (1.35–2.46)
**Street Connectivity**				
**1**	0.11	0.40	1.08	1.17
**2**	0.12	0.43	1.09	1.16
**3**	0.15	0.48	1.03	1.03
**4**	0.21	0.50	1.01	1.00
**5**	0.29	0.53	0.85	0.87
**Q1:Q5 Ratio (95% CI)**	0.38 (0.0–0.78)	0.74 (0.38–1.10)	1.27 (0.87–1.67)	1.36 (0.92–1.79)
**Destinations (within 800 m)**				
**1**	0.10	0.34	1.24	1.36
**2**	0.12	0.39	1.15	1.24
**3**	0.13	0.44	1.06	1.14
**4**	0.16	0.49	0.97	1.00
**5**	0.28	0.59	0.73	0.74
**Q1:Q5 Ratio (95% CI)**	0.36 (0.0–0.78)	0.58 (0.28–0.87)	1.70 (1.18–2.24)	1.84 (1.35–2.33)

*Transportation behaviors were derived from the Transportation Tomorrow Survey (2006) for residents age 11 and older.

**Walkability was calculated using data from: the 2006 Canada Census; DMTI Spatial Inc., 2009; the City of Toronto, 2009; and the Ministry of Education, 2009.

Individuals living in the lowest walkability areas had a 49.7% prevalence of overweight or obesity compared with 41.3% in the most walkable areas (Q1:Q5 ratio  = 1.18, 95% CI: 1.05–1.33). Similar relationships were found for each component of walkability, with a weaker and non-significant ratio for street connectivity (Table 3). The age-sex adjusted prevalence of diabetes among adults was 11.3% in the least walkable areas compared with 8.5% in the most walkable areas (Q1:Q5 ratio 1.33, 95% CI: 1.33–1.33), with consistent relationships found for each walkability component.

**Table 3:** Prevalence of Overweight*, Obesity,* and Diabetes** by Quintiles of Walkability and its Components*** for adults aged 30–64 years.

Quintile (1 = lowest; 5 = highest)	Overweight or Obese (%) 25) * ***(BMI> = 25)	Obese (%)	Diabetes Mellitus (%)
**Walkability Index**			
**1**	49.7	12.5	11.3
**2**	49.7	15.0	11.4
**3**	47.2	15.7	10.9
**4**	45.2	12.1	9.3
**5**	41.8	9.4	8.5
**Q1:Q5 ratio (95% CI)**	1.18 (1.05–1.33)	1.34 (0.96–1.71)	1.33 (1.33–1.33)
**Walkability Index Components**			
**Population Density**			
**1**	55.6	14.3	10.7
**2**	45.8	14.4	10.6
**3**	45.8	14.0	11.3
**4**	46.2	12.7	10.1
**5**	42.3	9.9	9.2
**Q1:Q5 ratio (95% CI)**	1.31 (1.16–1.47)	1.44 (1.02–1.85)	1.16 (1.16–1.16)
**Residential Density**			
**1**	52.3	14.1	11.3
**2**	46.1	14.1	10.9
**3**	47.4	14.4	11.4
**4**	47.9	12.6	9.9
**5**	41.5	9.9	8.5
**Q1:Q5 ratio (95% CI)**	1.26 (1.11–1.41)	1.42 (1.01–1.83)	1.33 (1.33–1.33)
**Street Connectivity**			
**Lowest = 1**	48.4	13.2	11.6
**2**	47.2	14.5	10.9
**3**	44.7	14.5	9.9
**4**	47.6	11.3	8.8
**Highest = 5**	43.5	9.2	8.4
**Q1:Q5 ratio (95% CI)**	1.11 (0.97–1.26)	1.43 (0.97–1.89)	1.38 (1.38–1.38)
**Destinations (within 800m)**			
**1**	48.2	12.9	11.0
**2**	47.8	13.7	10.8
**3**	49.4	14.1	11.1
**4**	47.1	14.4	9.9
**5**	41.4	9.6	8.7
**Q1:Q5 ratio (95% CI)**	1.16 (1.02–1.30)	1.34 (0.94–1.74)	1.26 (1.26–1.26)

*Overweight or obese (Body Mass Index > = 25) and obese (Body Mass Index > = 30) were derived from 2003–2008 Canadian Community Health Survey data.

**Age-sex adjusted prevalence of diabetes mellitus was derived from the Ontario Diabetes Database, 2009.

***Walkability was calculated using data from: the 2006 Canada Census; DMTI Spatial Inc., 2009; the City of Toronto, 2009; and the Ministry of Education, 2009.

**Figure 2:**
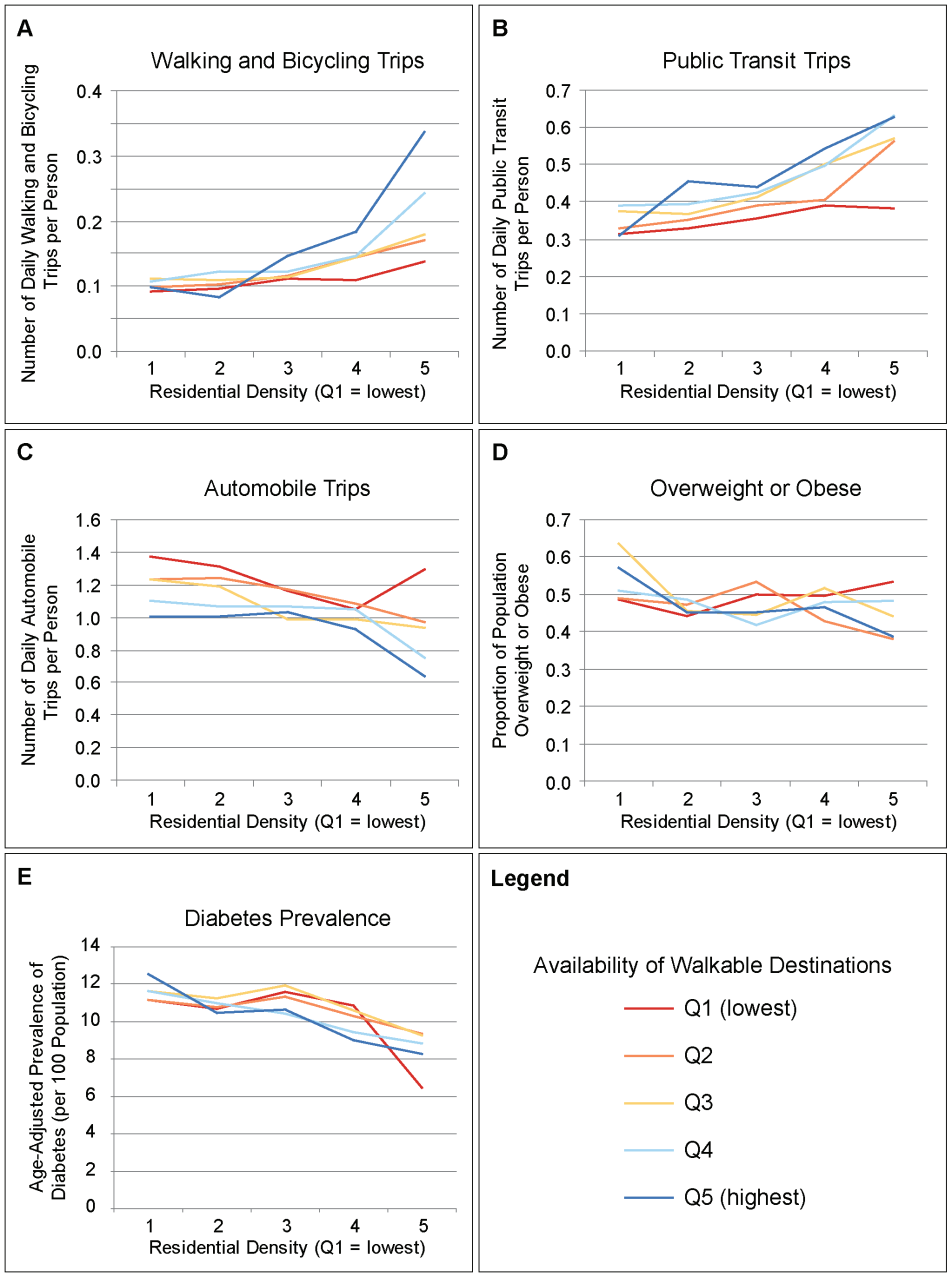
Transportation Behaviors and Health Outcomes by Residential Density and Availability of Walkable Destinations. These figures depict the interaction between density and destinations in relation to transportation behaviours and related health outcomes. The average daily number of trips per person by walking or bicycling, public transit, and automobile were derived from the Transportation Tomorrow Survey (2006) for residents age 11 years and older. Proportion of the population aged 30 to 64 years that were overweight and obese was derived from 2003–2008 Canadian Community Health Survey data. Age-sex adjusted prevalence of diabetes mellitus among adults aged 30 to 64 years was derived from the Ontario Diabetes Database, 2009. Residential density was calculated using data from the 2006 Canada Census and availability of walkable destinations was calculated using data from DMTI Spatial Inc. (2009), the City of Toronto (2009), and the Ministry of Education (2009).

Figure 2 (A–E) graphically presents the interaction analysis results that examined the effects of different levels of residential density and walkable destinations on our outcomes. The average number of daily walking and bicycling trips per person was low for all areas with low levels of residential density, regardless of the availability of walkable destinations. Walking and bicycling trips per person were consistently higher in areas with high residential density, with the highest levels of walking and bicycling found in those areas that had both a large number of walkable destinations and high residential density (p-value for interaction <0.0001). Within the highest quintile of residential density, the average number of daily walking and cycling trips per person was more than twice as high in the areas that also had the highest number of destinations as compared to the areas with the lowest number of destinations (0.34 vs. 0.14 trips per person). For automobile trips (p<0.0001), public transit trips (p<0.0026), and diabetes prevalence (p<0.0001) relationships with residential density and walkable destinations were also apparent, with the areas that had both high density and high destinations generally having the most favorable outcomes (Figure 2B,C and E). The interaction between residential density and walkable destinations was not significant for overweight or obesity.

## Discussion

This study found that both residential density and availability of walkable destinations had strong and consistent associations with transportation behaviors, overweight or obesity and diabetes. High residential density and availability of destinations tended to co-exist spatially; similarly, the absence of density and destinations also tended to occur together. Relatively few areas had only high density without many destinations or vice versa. Interestingly, the combination of high density and many walkable destinations had especially strong associations with transportation behaviors as compared to when only one was present, with similar and consistent findings for automobile trips and public transit trips. Overweight or obesity and diabetes followed a similar general pattern, though results for overweight or obesity did not reach statistical significance likely due to a small sample size. These findings suggest that either destinations or density could be used on their own as measures of walkability and related health outcomes in major urban settings such as Toronto, but that the combination of both variables may be most useful for identifying areas that are conducive to active transportation.

These findings advance the literature in several ways. Firstly, this study builds on existing findings through examining the role of density and destinations in a diverse urban context with high variation in population and residential densities and active transportation behaviors. Secondly, this study examines the individual and combined influence of density and destinations on diabetes using a validated population-based database and on overweight and obesity using data from a national health survey. We used maps and graphs to provide a comprehensive and transparent examination of interactions between walkability components, transportation behaviours and health outcomes. To our knowledge, no other study has examined similar relationships with health outcomes related to active transportation using population-based data of this quality and sample size.

One multi-city study examined associations between density and retail destinations and walking duration among middle- to older-age adults [31]. Results indicated consistent and significant associations between density and walking. A consistent increase in probability of walking was also found as density and retail destinations jointly increased, however the results did not reach statistical significance and the study may have had limited power due to the large number of categories used. By comparison, we found significant positive interactions between both density and destinations in relation to walking and bicycling trips, with stronger relationships found when both were present in an area. Another study conducted in Atlanta, Georgia using self-reported travel survey data found that study participants living in higher density neighborhoods were more likely to walk if the neighborhood also had more destinations to walk to and well-connected streets [30]. Results for obesity were more variable, with similar relationships found among some population subgroups and inverse relationships for others [30]. The authors note that the study area had limited variability in built environment characteristics, which may in part explain this finding.

This study has several strengths but should be interpreted in light of some limitations. The study was population-based using the smallest geographic areas possible in available data. It brought together data from a variety of sources including two population-based surveys, the Canada Census and a validated measure of diabetes in administrative data in a single payer system. The study further examined a previously-used walkability measure that was shown to have strong relationships with income, immigration, and diabetes [33]. We only examined a single municipality, a large diverse Canadian city, and as such applying these results elsewhere should be done cautiously. In particular, we found that residential density and availability of walkable destinations tended to co-exist spatially in our study area; the interaction effect between the two may differ in a setting where many areas have high density but have few destinations, or vice versa. It is also possible that areas with high density and destinations in our setting possess other design characteristics that support walking, such as higher intersection density, pedestrian-friendly design, and a mix of land uses. Due to the observational design we were unable to isolate the effect of density and destinations independent of these other factors and it is possible that density may act as a proxy for them [34]. Additionally, the study was cross-sectional and therefore describes correlations but should not be used to draw causal inferences. For example, it is not possible to tell from these data if people walk more because they live in areas that are more walkable versus whether people who choose to walk move to these sorts of areas. Reviews continue to cite the problem of self-selection as one of the leading limitations of built environment research [14], [16], yet experimental studies are difficult to mount and few longitudinal studies have been undertaken. Finally, in some cases, such as with the Census data, we were limited by the years available and we did not have individual level variables available for all of the measures used, precluding adjustment for individual-level factors such as age, sex, ethnicity or socioeconomic status. Those analyses should be the focus of future research.

In our urban setting, both density and destinations can be used to measure active transportation behaviors. If these relationships are similar in other settings, those wishing to understand and plan for walkable neighborhoods could use the data sources that were most readily available to them and expect to find consistent relationships with transportation behaviors and health outcomes. For example, census data that include residential density and population density are available to most decision-makers and planners and are likely an excellent starting point for understanding urban walkability. We did find advantages in combining measures of destinations and density, so using both types of measures in combination may add explanatory power and might be recommended when both types of data sources are available. These combinations could take the form of multi-dimensional walkability indices; however simple combinations such as the use of quintiles and maps, as conducted in this paper, could also be recommended.

We conclude that walkable urban environments may be important for stemming the tide of physical inactivity, overweight or obesity and diabetes and that walkability can be measured using either the availability of walkable destinations or residential density. In our setting, the combination of destinations and density provided additional explanatory power, a finding that should be examined in other settings.

## Supporting Information

Text S1 **Definition of Walkable Destinations.**
(DOC)
