# Correction: Density, Destinations or Both? A Comparison of Measures of Walkability in Relation to Transportation Behaviors, Obesity and Diabetes in Toronto, Canada

**DOI:** 10.1371/journal.pone.0091485

**Published:** 2014-03-14

**Authors:** The PLOS ONE Staff

In the author list, author Vered Kaufman Shriqui is listed incorrectly. The correct name is Vered Kaufman-Shriqui.
