# Supplementary material for: Density, Destinations or Both? A Comparison of Measures of Walkability in Relation to Transportation Behaviors, Obesity and Diabetes in Toronto, Canada
Source: PLoS One. 2014 Jan 14;9(1):e85295. doi: 10.1371/journal.pone.0085295 (PMC3891889; doi:10.1371/journal.pone.0085295)
Supplement: Text S1 — Definition of Walkable Destinations. (DOC) [file pone.0085295.s001.doc]

**Text SI. Definition of Walkable Destinations.**

Categories of Walkable Destinations were defined based on the LEED for Neighborhood Development list of “Diverse Uses” in Table S2 [1]. The SIC codes in Table S3 were used to query an initial selection of retail and service destination data from DMTI Spatial Inc. Enhanced Points of Interest data (2009). Records whose SIC description contained the terms ‘'Auto & home supply stores' or 'Hotels & Motels' or 'Household Appliance Stores' or 'Lumber & other building materials' or 'Paint, glass & wallpaper stores' and whose business name did not contain the terms 'CANADIAN TIRE' or 'HARDWARE' were excluded. This query was combined with public recreation center data from the City of Toronto (2009) and public elementary and secondary school data from the Ministry of Education (2009) to create the final walkable destinations dataset. Data from the Ministry of Education was provided for informational purposes only. Although the Ministry of Education endeavours to keep the information accurate and current, it cannot be held responsible for any damage resulting from its use.

**References**

[1] Congress for the New Urbanism; Natural Resources Defense Council; US Green Building Council (2012) LEED 2009 for Neighbourhood Development Rating System. Washington, DC: US Green Building Council. Available: [http://www.usgbc.org/sites/default/files/LEED%202009%20Rating_ND_10-2012_9c.pdf](http://www.usgbc.org/sites/default/files/LEED 2009 Rating_ND_10-2012_9c.pdf)
